# Noninferiority and Equivalence Evaluation of Clinical Performance among Computed Radiography, Film, and Digitized Film for Telemammography Services

**DOI:** 10.1155/2016/3642960

**Published:** 2016-09-29

**Authors:** Antonio J. Salazar, Javier A. Romero, Oscar A. Bernal, Angela P. Moreno, Sofía C. Velasco, Xavier A. Díaz

**Affiliations:** ^1^Electrophysiology and Telemedicine Laboratory, University of Los Andes, Carrera 1 Este No. 19A-40, Bogotá 11001, Colombia; ^2^Department of Diagnostic Imaging, Fundación Santa Fe de Bogotá University Hospital, Calle 119 No. 7-75, Bogotá 11001, Colombia; ^3^School of Medicine, University of Los Andes, Carrera 1 Este No. 19A-40, Bogotá 11001, Colombia; ^4^School of Government, University of Los Andes, Carrera 1 Este No. 19A-40, Bogotá 11001, Colombia

## Abstract

*Objective. *The aim of this study was to evaluate and compare the clinical performance of different alternatives to implement low-cost screening telemammography. We compared computed radiography, film printed images, and digitized films produced with a specialized film digitizer and a digital camera.* Material and Methods.* The ethics committee of our institution approved this study. We assessed the equivalence of the clinical performance of observers for cancer detection. The factorial design included 70 screening patients, four technological alternatives, and cases interpreted by seven radiologists, for a total of 1,960 observations. The variables evaluated were the positive predictive value (PPV), accuracy, sensitivity, specificity, and the area under the receiver operating characteristic curves (AUC).* Result*. The mean values for the observed variables were as follows: accuracy ranged from 0.77 to 0.82, the PPV ranged from 0.67 to 0.68, sensitivity ranged from 0.64 to 0.74, specificity ranged from 0.87 to 0.90, and the AUC ranged from 0.87 to 0.90. At a difference of 0.1 to claim equivalence, all alternatives were equivalent for all variables.* Conclusion.* Our findings suggest that telemammography screening programs may be provided to underserved populations at a low cost, using a film digitizer or a digital camera.

## 1. Introduction

Screening mammography programs, especially programs that use modern digital techniques such as computed radiography (CR) or full-field digital mammography (FFDM), have reduced the mortality rate associated with breast cancer [1, 2]. However, screening programs alone are inconclusive as they yield many false positives, and the definitive diagnosis of breast cancer is verified by biopsy and a histopathological examination of palpable lesions [3]. Therefore, the positive predictive value (PPV) of specific mammographic findings has been evaluated in several studies [4–6] and recently by Venkatesan et al. [7]. Nevertheless, the evaluation of sensitivity is also very important in the evaluation of mortality associated with false negatives.

Telemedicine may help to provide widespread screening mammography services in underserved areas, and approaches such as CR or FFDM are useful in the implementation of telemammography. However, these technologies are still unaffordable in vulnerable areas of our country, such as jungles that have a low population density; therefore, low-cost solutions are required for effective telemammography. In our country, CR is only available in large cities and FFDM is only available in our hospital. Specialized equipment is available for digitizing mammogram films, and several studies have compared digital mammogram modalities to film-screen mammography [8–10], reporting no significant differences between film-screen mammography and digital mammography modalities, such as CR and FFDM [11, 12]. Nevertheless, the cost of specialized digitizers is high, which is why low-cost alternative digitization equipment, such as conventional scanners and digital cameras, is being used for teleradiology services in developing countries. While such pieces of equipment can dramatically reduce costs, their clinical performance should be determined before introducing them in telemammography.

The aim of this study was to establish and to compare the clinical performance of different alternatives to implement telemammography, such as CR, film printed from the CR, a specialized digitizer, and a digital camera. The variables used for evaluating clinical performance were the PPV, sensitivity, specificity, accuracy, the area under the receiving operating characteristic (ROC) curve (AUC), and the proportions of true negatives (TN), true positives (TP), false negatives (FN), and false positives (FP), all of which were based on the final assessment categories of the Breast Imaging Reporting and Data System (BI-RADS) [13].

No significant differences between the compared modalities have been reported in other studies designed to test the null hypothesis that the performances of different modalities are equal, but the power test was not reported, so it is not clear if these studies failed to find significant differences. In statistical hypothesis testing, failure to reject the null hypothesis does not mean the null hypothesis is true. In contrast, the present study is set to evaluate equivalence or noninferiority, in which we can conclude equivalence or noninferiority based on significant results. To establish that the performances are equal or that one modality is noninferior to the other, the null hypothesis has to be that their performances are not equal or that one is inferior to the other. Only by rejecting such a hypothesis can we conclude that the modalities under comparison are equivalent [14–17].

## 2. Materials and Methods

The ethics committee of our institutions approved this retrospective study, and informed consent was not required. A factorial design with repeated measures was used in this study. The design of this study applied a treatment-by-reader-by-case factorial design with 70 patients, seven radiologists, three derived images, and the reference images (i.e., CR), for a total of 1,960 observations for each variable.

### 2.1. The Reference Standard

The standard for positive cases was a malignant lesion confirmed by biopsy within two years of the initial mammography screening, corresponding to BI-RADS final assessment categories 4A, 4B, 4C, and 5 [9, 12, 18]. Negative cases were defined as cases without any lesions confirmed by biopsy or cases with normal follow-up mammograms within the same two-year interval, corresponding to BI-RADS final assessment categories 2 and 3. Two radiologists with more than ten years of experience in reading mammograms who had access to the clinical history of the patients (biopsy, follow-up mammograms, etc.) established the reference standard.

### 2.2. Study Sample and Readers

At most rural health centers in our country, there are no mammography services [19], and where they are available, there are no mammograms repositories, so there are not available mammograms to use for a retrospective study. In addition, in these regions, there are not enough patients to develop a prospective study in a short time. For these reasons, this study was undertaken using CR screening mammograms from our hospital, which is a reference hospital for mammography screening, serving patients from remote undeserved areas of our country (approximately 8,000 mammograms interpreted per year). Mammography studies from patients who attended mammography screenings at the Fundación Santa Fe de Bogotá University Hospital (FSFBUH) were randomly selected without repetition from our screening database; the patients were all asymptomatic, and their lesions were impalpable and verified by pathology. The masses ranged in size from 6 mm to 23 mm, with a mean of 11 mm (SD = 4.2). Each case was required to include the following four standard mammographic views: mediolateral oblique, craniocaudal, left, and right, even if additional views were taken in the original screening mammograms. Cases of tomosynthesis or large masses were excluded.

To determine the sample size, we used the table proposed by Obuchowski [20] for comparisons of the AUC with the following criteria: (a) six observers, (b) small variability between radiologists, (c) moderate accuracy of the test (an AUC of approximately 0.75), (d) moderate differences suspected to be found among AUC (i.e., *δ* = 0.1), and (e) a 1 : 1 ratio between malignant and benign cases. Using these criteria, minimum 60 cases were required. The final sample size was set at 70 cases, and the number of radiologists was increased to seven. Patients ranged in age from 41 to 84 years, with a mean age of 62.1 years (SD = 11.5). The cases were distributed as follows: 33 patients had cancer and 37 patients had benign lesions or normal results. The distribution of cases according to the BI-RADS final assessment categories is shown in Table 1. There were 57 cases with calcifications, 26 with masses, 35 with asymmetries, and 11 with architectural distortions and associated features. Four patients with prostheses were included in the sample. The detailed lesion classification of the cases is presented in Table 2. In terms of composition, the distribution of cases was as follows: 17 of the breasts were almost entirely fatty, 32 had scattered areas of fibroglandular density, 11 of the breasts were heterogeneously dense, which may obscure small masses, and 10 of the breasts were extremely dense, which lowers the sensitivity of mammography.

Seven radiologists from FSFBUH who were experienced in mammography, including four with high levels of experience (more than 10 years) and three with intermediate levels of experience (more than two years), served as observers.

### 2.3. Variables Observed by the Radiologists

Data collection was performed using a database and a digital form that was integrated into the image viewing software. At each interpretation, the radiologist selected the level of confidence in the presence of each selected condition, that is, calcifications, nodules, asymmetries, and distortions, from the following scores: 0, definitely absent; 1, most likely absent; 2, cannot decide; 3, most likely present; and 4, definitely present. For conditions with scores of 3 or 4, the radiologist was required to classify the condition according to the value in Table 2. Next, the radiologist classified the breast composition and finally at the conclusion of this process, a BI-RADS final assessment category was selected.

### 2.4. Generation and Digitization of the Mammograms

**Figure 1:**
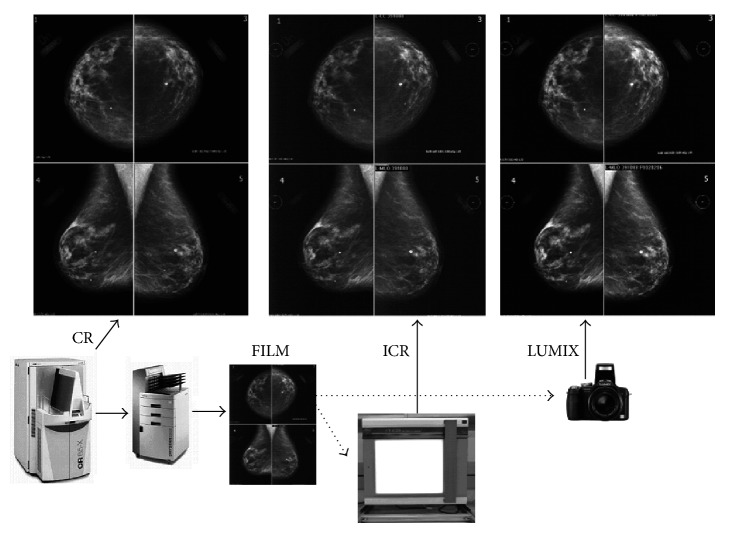
Digital image and film generation. CR: computed radiography; FILM: printed film; LUMIX: Lumix DMC-FZ28 digital camera; ICR: iCR 612SL specialized digitizer.

The process of generating film and digital images is shown in Figure 1. The original mammograms consisted of screening CR images that were stored in the picture archiving and communication system (PACS) at FSFBUH. Routine screening digital mammograms were acquired using an Agfa CR 85-X (Agfa HealthCare NV, Belgium), hereafter referred to as CR, with a resolution of 20 pixels/mm (508 dpi), 50 *μ*m per pixel, and a 14-bit grayscale from an 18 × 24 cm chassis and a 3,560 × 4,640-pixel matrix. The derived mammogram images were generated as follows: as we had no screen-film images, the CR images were printed under the supervision of a radiologist on an 18 × 24 cm film with a digital Agfa Drystar 5503 printing system (Agfa HealthCare NV, Belgium) with a resolution of 508 dpi, 50 *μ*m per pixel, and 14-bit contrast. Data that could be used to identify patients were not included in the printed mammograms. Next, the films were digitized using the following two capture devices: (1) an iCR 612SL specialized digitizer (iCR Company, Torrance, CA) that had a maximum spatial resolution of 875 dpi, a pixel spot of 29 *µ*m, 16 bits per pixel, an optical density (OD) of 3.6, and a cost of $15,000 (hereafter referred to as ICR) and (2) a Lumix DMC-FZ28 digital camera (Panasonic Corporation, Secaucus, NJ, USA) with a 10-megapixel resolution, a focal length of 4.8 to 86.4 mm, a 1/2.33′′ charge-coupled device (CCD), ISO 100–6,400, and a cost of $450 (plus $400 for support system and light box). The digital camera is hereafter referred to as LUMIX.

**Figure 2:**
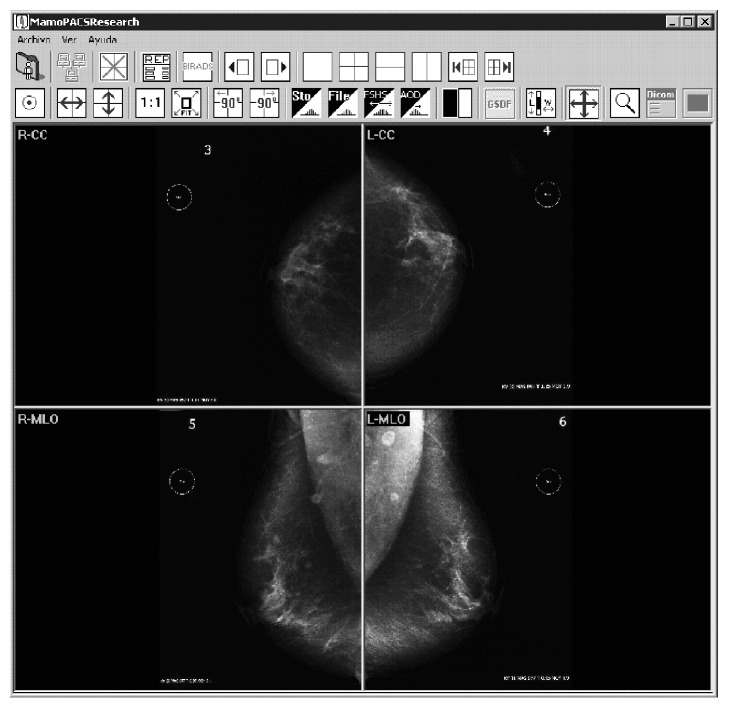
Interpretation software. This software is compliant with the Digital Imaging and Communication in Medicine (DICOM) standard.

For each patient (case), the following four case studies were obtained: (1) the printed film, hereafter referred to as the FILM, and three images in digital form, including (2) images from the CR (3,560 × 4,640-pixel matrix and 14-bit grayscale), (3) images digitized with the ICR (2,436 × 3,636-pixel matrix and 8-bit grayscale), and (4) images digitized with the LUMIX (2,538 × 3,463-pixel matrix and 8-bit grayscale). This procedure was completed for each of the 70 sample mammograms, producing 280 case studies. DICOM-compliant software that was developed at our institution and previously tested in several studies [21–24] was used to scan, store, and display the cases (see Figure 2).

### 2.5. Display

At a cost of $8,500, a DICOM-compliant 3-MPixel MD213MG (NEC Display Solutions, Tokyo, Japan) medical-grade grayscale display, with a dot pitch of 0.21 mm, a spatial resolution of 2,048 × 1,536 pixels, maximum luminance of 1,450 cd/m^2^, and 10-bit grayscale (i.e., 1,024 gray levels), was used as the display monitor.

### 2.6. Data Analysis

To compare the AUC for the detection of patients with cancer, analyses of variance (ANOVA) of the pseudovalues of the AUC were performed using DBM-MRMC 2.3 software [21]. Using the BI-RADS final assessment category as the endpoint variable, we classified all readings as negative (BI-RADS, 2 and 3) or positive (BI-RADS, 4A, 4B, 4C, and 5) [9, 12, 18], and we calculated contingency tables for these values, that is, the total true positives (tTP), the total true negatives (tTN), the total of false positives (tFP), and the total of false negatives (tFN). The common diagnostic metrics were calculated for these variables as follows: PPV = tTP/(tTP + tFP), sensitivity = tTP/(tTP + tFN), accuracy = (tTP + tTN)/(total sample), specificity = tTN/(tTN + tFP), and the area under the receiving operating characteristic (ROC) curve (AUC). In addition, we calculated the proportions of true positives TP = tTP/(total sample), the proportions of true negatives TN = tTN/(total sample), the proportions of false positives FP = tFP/(total sample), and the proportions of false negatives FN = tFN/(total sample).

These variables and the difference between the compared modalities were evaluated using generalized estimating equations (GEE) with the IBM SPSS Statistics 19 software (IBM Corp., Armonk, NY, USA). With the purpose of evaluating noninferiority and equivalence, the mean differences and their standard errors were obtained from DBM-MRMC and SPSS software.

The hypothesis test for equivalence was as follows: the null hypothesis Ho was |Mean  Difference  (*I* − *J*)| − *δ* = 0 and the alternative hypothesis Ha was |Mean  Difference  (*I* − *J*)| − *δ* < 0, where *I* and *J* are the two modalities compared and *δ* (delta) is the maximum allowable difference permitted to conclude equivalence or noninferiority, as suggested by several authors in recent years [14–17]. We calculated a (1-2*α*)% confidence interval for all comparisons, which is a method to evaluate equivalence [16, 17]. The significance level was set to 5% (i.e., *α* = 0.05) and *δ* was set to 0.1, as this was the difference established in the sample selection to evaluate the area under the ROC curves. We were interested in evaluating equivalence using lower values for *δ*, in particular *δ* = 0.05, to assess the PPV and sensitivity for screening purposes. Finally, we calculated the required value of *δ* to claim equivalence for each variable and the comparison.

### 2.7. Procedure

Each radiologist read each case using the following viewing methods: the film in a light box and three viewings on the medical display for digital cases of CR, ICR, and LUMIX. Pairs of patients and devices were presented at random by the software; hence, there were at least 30 different patients before a patient was repeated for any radiologist. At each reading, the radiologist determined the variables mentioned in the section entitled “Observed Variables.” Each radiologist received training in the use of the viewer software before the readings were initiated. A pilot study was conducted to determine the usefulness of the viewer software and the interpretation form. The software provides case blinding and several image manipulation tools to adjust the window/level, brightness, and contrasts and histogram tools (e.g., the average optical density, histogram equalization, and full-scale histogram stretching). These tools may be combined with the overall zoom and the magnifying glass. These tools were available for all images and could be used at the observer's discretion to improve image quality, especially for patients with dense breasts and amorphous calcifications. The readings were performed over the course of ten months in two- or four-hour sessions by each radiologist, with no time limitations for each reading.

## 3. Results 

### 3.1. Mean Values by Device

The mean values, standard error of the mean, and the 95% confidence interval for each device and each calculated variable presented in the data analysis section are shown in Table 3. Each of these means was calculated from 490 observations (70 cases and seven radiologists). The TN ranged from 0.46 to 0.48, the TP ranged from 0.30 to 0.35, the FN ranged from 0.12 to 0.17, and the FP ranged from 0.05 to 0.07. The mean values for the derived variables were as follows: accuracy ranged from 0.77 to 0.82, the PPV ranged from 0.67 to 0.68, sensitivity ranged from 0.64 to 0.74, specificity ranged from 0.87 to 0.90, and the AUC ranged from 0.87 to 0.90.

### 3.2. Mean Difference Values and the Equivalence Test by Paired Devices in Proportion Variables

The mean values of the differences and equivalence tests for the TN, TP, FN, and FP by paired devices are shown in Table 4 (for *δ* = 0.1) and Table 5 (for *δ* = 0.05). The mean values of the differences and the equivalence tests for accuracy, the PPV, sensitivity, specificity, and the AUC by paired devices are shown in Table 6 (for *δ* = 0.1) and Table 7 (for *δ* = 0.05). For both Tables 4 and 6, the equivalence test was preformed using *δ* = 0.1 as the original setting of this study in terms of the AUC, and in addition, a value of *δ* = 0.05 was included as explained previously. In the last column of both Tables 5 and 7, the calculated value of *δ* required in each variable and comparison to conclude equivalence between the compared devices is presented.

The absolute differences for the calculated variables were as follows: the TN differences ranged from 0.000 to 0.016, the TP differences ranged from 0.008 to 0.047, the FN differences ranged from 0.008 to 0.047, and the FP differences ranged from 0.000 to 0.016. For *δ* = 0.1, all the comparisons in Table 4 showed equivalence (*P* < 0.001); for *δ* = 0.05, most comparisons (20) showed equivalence (*P* values ranged from 0.0001 to 0.0347), while no significant differences were found for the TP and the FN in LUMIX versus FILM and ICR versus FILM; nevertheless, the required *δ* to achieve equivalence was near 0.05 (0.051 and 0.069).

### 3.3. Mean Difference Values and the Equivalence Test by Paired Devices for the Derived Variables

The absolute differences for the derived variables were as follows: the accuracy differences ranged from 0.010 to 0.057, the PPV differences ranged from 0.002 to 0.009, the sensitivity differences ranged from 0.017 to 0.100, the specificity differences ranged from 0.000 to 0.031, and the AUC differences ranged from 0.009 to 0.034. For *δ* = 0.1, all the comparisons for accuracy, the PPV, and specificity showed equivalence (*P* values ranged from 0.0001 to 0.004), while for sensitivity, again, the LUMIX-FILM and ICR-FILM comparisons showed no significant differences.

For *δ* = 0.1 in the AUC tests, the comparisons showed statistical equivalence for the following pairs: LUMIX versus CR (*P* = 0.008), LUMIX versus FILM (*P* = 0.04), and FILM versus CR (*P* = 0.03); in the LUMIX versus ICR comparison, equivalence was not found, but the noninferiority of LUMIX was observed (*P* = 0.046); for ICR versus CR and ICR versus FILM, neither equivalence nor noninferiority was noted, and the required values of *δ* to achieve equivalence were 0.133 and 0.118, respectively. However, for *δ* = 0.05, less consistency was observed. Only paired comparisons for the PPV were all equivalent (*P* < 0.001); for specificity, the LUMIX versus FILM comparison failed to show equivalence (*P* = 0.15). For *δ* = 0.05, in paired comparisons for the other derived variables, few tests confirmed equivalence: three showed equivalent accuracy, three showed equivalent sensitivity, and only one showed an equivalent AUC.

In general, the required values for *δ* to confirm equivalence ranged from 3.4% to 8.4% for accuracy, 0.7% to 1.5% for the PPV, 6.8% to 14.2% for sensitivity, 3.8% to 6.1% for specificity, and 7.3% to 13.3% for the AUC.

### 3.4. Evaluations of Dense Breasts

We ran the GEE analysis using only the readings of cases with heterogeneously dense and extremely dense (21 patients by 7 radiologists: 147 interpretations) breasts for the TP, TN, FP, FN, VPP, sensitivity, specificity, and accuracy evaluations (see Table 8). The best values of these variables were observed for FILM; nevertheless, the values for the digital images were very similar regardless of whether the device is of highest or lowest resolution, that is, CR or LUMIX, respectively. In pairwise comparisons between the high-resolution device (CR) and the low-resolution devices (ICR and LUMIX), the results were as follows: between CR and ICR, no significant differences were observed for the TP, TN, FP, FN, VPP, VPN, sensitivity, specificity, and accuracy; between CR and LUMIX, no significant differences were observed for the TP, TN, FP, FN, sensitivity, specificity, and accuracy. In pairwise comparisons between printed film (FILM) and the three digital devices (CR, ICR, and LUMIX), the results were as follows: no significant differences were found for the TP and TN, nor for the FP, FN, VPN, specificity, and accuracy, while differences were noted for the sensitivity and VPP between FILM and CR. In comparisons between FILM and ICR and LUMIX, which are digital images with lower resolutions, differences were noted in the TP, FN, VPP, VPN, sensitivity, accuracy, and VPP, and for the specificity between FILM and LUMIX; while no differences were observed for the TP, FP, and VPN. High values for the AUC (ranging from 0.86 to 0.90), with no significant differences, were found among the four devices (*P* = 0.186).

As we found many nonsignificant differences (*P* > 0.05), we performed equivalence analyses, finding *δ* (delta) values for which equivalence may be claimed with significant values. In this analysis, LUMIX and CR achieved TP equivalent at 4%, while ICR and CR achieved TP equivalent at 2.2%. The TN were equivalent at 7.3% for CR-LUMIX and 6.1% for CR-ICR. Sensitivities were equivalent at 6.5% for CR-LUMIX and 3.6% for CR-ICR. The VPP values were equivalent at 7.5% for CR-LUMIX and 1.8% for CR-ICR. Only for specificity comparisons were the equivalence values larger than 10%. In this analysis, LUMIX and CR achieved AUC values equivalent at 4.9%, while ICR and CR achieved AUC values equivalent at 7.3%. Compared to FILM, AUC values of LUMIX and ICR were equivalent at 3.8% and 5.2%, respectively, while CR was equivalent at 6.8%.

### 3.5. The Evaluation of Amorphous Calcifications

To evaluate this point, we ran the GEE analysis using only the readings of cases with amorphous calcifications (7 patients evaluated by 7 radiologists, with a total of 49 interpretations for each device) for the TP, TN, FP, FN, VPP, sensitivity, specificity, and accuracy. There were no true negatives nor false positives, and thus the mean value of the VPP was 1.0 and the sensitivity, TP, FN, and accuracy values were all equal to 0.63, while the VPN, specificity, TN, and FP were 0.0. There were no significant differences in the sensitivity, TP, and FN (*P* = 0.133). The results of the comparisons of TP for LUMIX and ICR versus CR (i.e., the original reference image) were as follows: a larger TP mean for LUMIX (0.65) compared to CR (0.63), but with no significant difference (*P* = 0.053). The CR was greater than the ICR, but again with no significant difference (*P* = 1.0). The results of comparisons of the TP for LUMIX and ICR versus FILM (which is a derived image printed from the original CR) were as follows: larger TP were observed for FILM (0.71) but with no significant differences among CR, ICR, or LUMIX (this is an expected result, as the overall analysis was not significant). As we found no significant differences (*P* > 0.05), we performed equivalence analyses, finding *δ* values for which equivalence may be claimed with significant values. In this analysis, the LUMIX was equivalent to CR at 10.8% and ICR was equivalent to CR at 14.1%. With respect to the printed FILM, *δ* (delta) value was lower for LUMIX (14%) while the CR (the original and the larger digital image) delta value was 19.7%.

## 4. Discussion 

The values observed for the AUC for each device ranged from 0.87 to 0.90. These accuracies were higher than the assumed value accuracy used in the sample size calculation for this study (i.e., 0.75). In the paired comparisons, low differences were observed for most derived variables; for PPV, which is one of the most important variables in mammography [4–6], all values were inferior to 0.9% (0.009). In contrast, the largest differences identified among the paired comparisons were 10.0% (0.1) for sensitivity in a comparison of ICR and FILM. Readings from the LUMIX, which was the lowest-cost device in this study, were equivalent to CR in terms of accuracy, the PPV, sensitivity, specificity, and the AUC for *δ* = 0.1. This is important because the LUMIX images were obtained after printing CR images on film and digitizing them with the camera, which may deteriorate the quality of these images. Comparing LUMIX with ICR (which is approximately 30 times more expensive than LUMIX), equivalence was observed in terms of the accuracy, the PPV, sensitivity, specificity, and noninferiority in the AUC for *δ* = 0.1.

In this study, we used a value of *δ* = 0.1 (10%) to evaluate equivalence, which was the value used in this study and in our previous studies to calculate sample size [22, 25, 26]. With this value, global equivalence was observed. As a post hoc evaluation, *δ* = 0.05 was used to be more conservative with respect to sensitivity. With this value, fewer comparisons showed equivalence or noninferiority at a cutoff significance level of 0.05. The value of the required *δ* to achieve equivalence may be useful in further calculations of the required sample size for similar studies.

Our results regarding dense breasts suggest that the lower digital images of the digital camera LUMIX and especially ICR are still good quality low-cost alternatives, even for heterogeneously dense and extremely dense breasts, with better performance observed for ICR than LUMIX. The results provide support for the hypothesis that there are no significant differences between the interpretations of CR mammography examinations and soft copy examinations produced by a specialized film digitizer or a digital camera. In the same sense, our results suggest that the lower quality digital images of the digital camera LUMIX are still of adequate quality even for amorphous calcifications.

A limitation of our study, as explained before, is that all of the mammography images in this study were obtained from a referral hospital with high standards and quality equipment. Therefore, the results of this study should be revisited using film-screen mammography images obtained at rural hospitals with equipment and technical standards of varying quality. Another limitation of this study is the variability between radiologists. Consequently, it was more difficult to obtain significant results when less-than-10% non-inferiority or equivalence margins were selected. A third limitation was the selection procedure to establish this margin, which must be a predetermined clinically meaningful limit. The researchers of this study did not agree when to set the value at 5% or 10%, or another more appropriate value, for the inferiority or equivalence margin, and of course, this value may be different for each calculated variable (e.g., sensitivity, specificity, the PPV, and the AUC). This disagreement is due to ignorance regarding the actual values that these variables take on when our radiologists interpret routine mammograms. In this sense, this study is a first estimation of these values and can be used to improve sample size calculations in further studies at our hospital.

In our analysis, the specificity and AUC values were high, whereas the accuracy and PPV were moderate, and the sensitivity values were relatively low. Other studies have compared film-screen mammography with digitized film [8–10, 12] and reported no significant differences in their diagnostic accuracy, but these studies were not equivalence or noninferiority evaluations, and no report about the power test was presented. In our study, we measured mean AUC values that were similar to or higher than those reported by Powell et al. [8], Gitlin et al. [9], and Pisano et al. [10] and by Lewin et al. using FFDM [18]. To our knowledge, no previous study has evaluated the equivalence or noninferiority performance of observers reading mammograms that were captured with a digital camera.

Screening may have side effects associated with false positives. Previous studies have shown that one in three test results leads to biopsy, which often turns out to be negative for cancer. Even when cancer is ruled out by the pathology results, high rates of testing generate a 33% cost overrun for screening [27] and cause permanent anxiety in patients [1]. Moreover, 50% of cancer patients survive regardless of whether they were enrolled in a screening program [2]. The risk of false positives should be maintained below 10% by comparing successive screening mammograms at intervals of 12 to 18 months until the patient's life expectancy is less than 10 years [22]. In our study, low FP were noted (<6.7%) for all devices, which is important for reducing stress in patients and the health system costs.

The principal difference of our study with respect to previous studies is that in this evaluation an equivalence or noninferiority study was performed, instead of a conventional two-sided hypothesis test setting for the nonequivalence testing as was the case in many previously published articles, in which no statistical differences were reported without reporting the power test.

## 5. Conclusion

In conclusion, our findings suggest that telemammography screening programs may be provided to underserved populations at a low cost, using a film digitizer or a digital camera, with differences of 10% in terms of the sensitivity, specificity, positive predictive value, accuracy, and the area under the receiver operating characteristic curve. To increase the power in equivalence or noninferiority tests for margin differences of 5%, more images or more observers must be included in the study.

## Figures and Tables

**Table 1:** Distribution of cases in the sample according to the BI-RADS final assessment categories.

BI-RADS final assessment category^a^	Cases
2: benign	18
3: probably benign	19
4A: low suspicion for malignancy	6
4B: moderate suspicion for malignancy	14
4C: high suspicion for malignancy	3
5: highly suggestive of malignancy	10

Total	70

^a^Classification according to the American College of Radiology [13].

**Table 2:** Detailed classification of the cases in the sample.

Condition	Classification^a^	Cases
Masses	Well-defined mass	7
	Obscured edge mass	10
	Poorly defined mass	4
	Spiculated mass	5

Calcifications	Benign calcifications	33
	Solitary group of punctate calcifications	4
	Coarse heterogeneous calcification	8
	Amorphous calcification	7
	Fine pleomorphic calcifications	4
	Pleomorphic ductal pattern	1

Architectural distortions and associated features		11

Asymmetries	Asymmetry	23
	Focal asymmetry	12

^a^Classification according to the American College of Radiology [13].

**Table 3:** Mean values for the calculated variables by device.

Variable^a^	Device	Mean	SE	95% confidence interval	
				Lower	Upper
TN	CR	0.47	0.05	0.36	0.57
	ICR	0.47	0.05	0.36	0.57
	LUMIX	0.46	0.05	0.36	0.57
	FILM	0.48	0.06	0.37	0.59

TP	CR	0.32	0.05	0.23	0.42
	ICR	0.30	0.05	0.21	0.39
	LUMIX	0.32	0.05	0.22	0.41
	FILM	0.35	0.05	0.25	0.45

FN	CR	0.15	0.03	0.08	0.21
	ICR	0.17	0.04	0.10	0.24
	LUMIX	0.16	0.03	0.09	0.22
	FILM	0.12	0.03	0.06	0.18

FP	CR	0.06	0.02	0.03	0.09
	ICR	0.06	0.02	0.03	0.09
	LUMIX	0.07	0.02	0.03	0.10
	FILM	0.05	0.02	0.02	0.08

Accuracy	CR	0.79	0.03	0.73	0.86
	ICR	0.77	0.04	0.70	0.84
	LUMIX	0.78	0.03	0.72	0.84
	FILM	0.82	0.03	0.76	0.89

PPV	CR	0.68	0.06	0.57	0.79
	ICR	0.68	0.06	0.56	0.79
	LUMIX	0.67	0.06	0.56	0.79
	FILM	0.68	0.06	0.57	0.79

Sensitivity	CR	0.69	0.06	0.58	0.80
	ICR	0.64	0.06	0.52	0.76
	LUMIX	0.67	0.05	0.57	0.77
	FILM	0.74	0.06	0.63	0.84

Specificity	CR	0.88	0.03	0.83	0.94
	ICR	0.88	0.03	0.83	0.94
	LUMIX	0.87	0.03	0.82	0.93
	FILM	0.90	0.03	0.85	0.96

AUC	CR	0.89	0.04	0.81	0.97
	ICR	0.87	0.05	0.77	0.96
	LUMIX	0.88	0.04	0.80	0.96
	FILM	0.90	0.02	0.87	0.94

^a^Each mean was calculated from 490 observations (70 cases and seven radiologists). TN: true negative proportion, TP: true positive proportion, FN: false negative proportion, FP: false positive proportion, PPV: positive predictive value, ROC: receiver operating characteristic, AUC: area under ROC curve, SE: standard error of the mean.

**Table 4:** Equivalence tests (*δ* = 0.1) for TN, TP, FN, and FP by paired devices.

Variable^a^	Compared devices		Mean difference (*I* − *J*)	SE	(1-2*α*)% confidence interval for equivalence testing		*z*	*P*	*H*
	(*I*) device	(*J*) device			Lower	Upper			
TN	LUMIX	ICR	−0.006	0.0102	−0.023	0.011	−9.22	<0.001	Ha
	LUMIX	CR	−0.006	0.0084	−0.020	0.008	−11.20	<0.001	Ha
	LUMIX	FILM	−0.016	0.0098	−0.032	0.000	−8.53	<0.001	Ha
	ICR	CR	0.000	0.0091	−0.015	0.015	−10.96	<0.001	Ha
	ICR	FILM	−0.010	0.0097	−0.026	0.006	−9.25	<0.001	Ha
	FILM	CR	0.010	0.0067	−0.001	0.021	−13.49	<0.001	Ha

TP	LUMIX	ICR	0.016	0.0098	0.000	0.032	−8.53	<0.001	Ha
	LUMIX	CR	−0.008	0.0104	−0.025	0.009	−8.86	<0.001	Ha
	LUMIX	FILM	−0.031	0.0122	−0.051	−0.011	−5.68	<0.001	Ha
	ICR	CR	−0.024	0.0104	−0.042	−0.007	−7.26	<0.001	Ha
	ICR	FILM	−0.047	0.0135	−0.069	−0.025	−3.94	<0.001	Ha
	FILM	CR	0.022	0.0152	−0.003	0.047	−5.11	<0.001	Ha

FN	LUMIX	ICR	−0.016	0.0098	−0.032	0.000	−8.53	<0.001	Ha
	LUMIX	CR	0.008	0.0104	−0.009	0.025	−8.86	<0.001	Ha
	LUMIX	FILM	0.031	0.0122	0.011	0.051	−5.68	<0.001	Ha
	ICR	CR	0.024	0.0104	0.007	0.042	−7.26	<0.001	Ha
	ICR	FILM	0.047	0.0135	0.025	0.069	−3.94	<0.001	Ha
	FILM	CR	−0.022	0.0152	−0.047	0.003	−5.11	<0.001	Ha

FP	LUMIX	ICR	0.006	0.0102	−0.011	0.023	−9.22	<0.001	Ha
	LUMIX	CR	0.006	0.0084	−0.008	0.020	−11.20	<0.001	Ha
	LUMIX	FILM	0.016	0.0098	0.000	0.032	−8.53	<0.001	Ha
	ICR	CR	0.000	0.0091	−0.015	0.015	−10.96	<0.001	Ha
	ICR	FILM	0.010	0.0097	−0.006	0.026	−9.25	<0.001	Ha
	FILM	CR	−0.010	0.0067	−0.021	0.001	−13.49	<0.001	Ha

^a^Each comparison was calculated from 980 observations (70 cases, seven radiologists, and two devices). TN: true negative proportion, TP: true positive proportion, FN: false negative proportion, FP: false positive proportion, SE: standard error of the mean, Ho: null hypothesis, Ha: alternative hypothesis for testing equivalence, *α*: significance of the test (0.05), *δ*: difference of the means allowed to achieve equivalence, *z*: test for difference of compared devices, that is, *z* = (|Difference (*I* − *J*)| − *δ*)/SE, *H*: retained hypothesis equivalence at *δ* level (“Ha” indicates equivalence achieved and “Ho” indicates failing to reject the null hypothes).

Ho: |difference (*I* − *J*)| − *δ* = 0.

Ha: |difference (*I* − *J*)| − *δ* < 0.

**Table 5:** Equivalence tests (*δ* = 0.05) for TN, TP, FN, and FP by paired devices.

Variable^a^	Compared devices		(1-2*α*)% confidence interval for equivalence testing		*z*	*P*	*H*	*δ*_*e*_
	(*I*) device	(*J*) device	Lower	Upper				
TN	LUMIX	ICR	−0.023	0.011	−4.31	<0.001	Ha	0.023
	LUMIX	CR	−0.020	0.008	−5.23	<0.001	Ha	0.020
	LUMIX	FILM	−0.032	0.000	−3.43	<0.001	Ha	0.032
	ICR	CR	−0.015	0.015	−5.48	<0.001	Ha	0.015
	ICR	FILM	−0.026	0.006	−4.10	<0.001	Ha	0.026
	FILM	CR	−0.001	0.021	−5.98	<0.001	Ha	0.021

TP	LUMIX	ICR	0.000	0.032	−3.43	<0.001	Ha	0.032
	LUMIX	CR	−0.025	0.009	−4.04	<0.001	Ha	0.025
	LUMIX	FILM	−0.051	−0.011	−1.59	0.06	Ho	0.051
	ICR	CR	−0.042	−0.007	−2.45	0.007	Ha	0.042
	ICR	FILM	−0.069	−0.025	−0.23	0.41	Ho	0.069
	FILM	CR	−0.003	0.047	−1.82	0.03	Ha	0.047

FN	LUMIX	ICR	−0.032	0.000	−3.43	<0.001	Ha	0.032
	LUMIX	CR	−0.009	0.025	−4.04	<0.001	Ha	0.025
	LUMIX	FILM	0.011	0.051	−1.59	0.06	Ho	0.051
	ICR	CR	0.007	0.042	−2.45	<0.001	Ha	0.042
	ICR	FILM	0.025	0.069	−0.23	0.41	Ho	0.069
	FILM	CR	−0.047	0.003	−1.82	0.03	Ha	0.047

FP	LUMIX	ICR	−0.011	0.023	−4.31	<0.001	Ha	0.023
	LUMIX	CR	−0.008	0.020	−5.23	<0.001	Ha	0.020
	LUMIX	FILM	0.000	0.032	−3.43	<0.001	Ha	0.032
	ICR	CR	−0.015	0.015	−5.48	<0.001	Ha	0.015
	ICR	FILM	−0.006	0.026	−4.10	<0.001	Ha	0.026
	FILM	CR	−0.021	0.001	−5.98	<0.001	Ha	0.021

^a^Each comparison was calculated from 980 observations (70 cases, seven radiologists, and two devices). TN: true negative proportion, TP: true positive proportion, FN: false negative proportion, FP: false positive proportion, SE: standard error of the mean, Ho: null hypothesis, Ha: alternative hypothesis for testing equivalence, *α*: significance of the test (0.05), *δ*: difference of the means allowed to achieve equivalence, *z*: test for difference of compared devices, that is, *z* = (|Difference (*I* − *J*)| − *δ*)/SE, *H*: retained hypothesis equivalence at *δ* level (“Ha” indicates equivalence achieved and “Ho” indicates failing to reject the null hypothes).

Ho: |difference (*I* − *J*)| − *δ* = 0.

Ha: |difference (*I* − *J*)| − *δ* < 0.

**Table 6:** Equivalence tests for accuracy, PPV, sensitivity, specificity, and AUC by paired devices (*δ* = 0.1).

Variable^a^	Compared devices		Mean difference (*I* − *J*)	SE	(1-2*α*)% confidence interval for equivalence testing		*z*	*P*	*H*
	(*I*) device	(*J*) device			Lower	Upper			
Accuracy	LUMIX	ICR	0.010	0.0142	−0.013	0.034	−6.31	<0.001	Ha
	LUMIX	CR	−0.014	0.0133	−0.036	0.008	−6.46	<0.001	Ha
	LUMIX	FILM	−0.047	0.0152	−0.072	−0.022	−3.49	<0.001	Ha
	ICR	CR	−0.024	0.0138	−0.047	−0.002	−5.46	<0.001	Ha
	ICR	FILM	−0.057	0.0162	−0.084	−0.031	−2.65	0.004	Ha
	FILM	CR	0.033	0.0164	0.006	0.060	−4.11	<0.001	Ha

PPV	LUMIX	ICR	−0.003	0.0052	−0.011	0.006	−18.69	<0.001	Ha
	LUMIX	CR	−0.007	0.0049	−0.015	0.001	−18.79	<0.001	Ha
	LUMIX	FILM	−0.009	0.0050	−0.017	−0.001	−18.28	<0.001	Ha
	ICR	CR	−0.005	0.0047	−0.012	0.003	−20.32	<0.001	Ha
	ICR	FILM	−0.007	0.0040	−0.013	0.000	−23.08	<0.001	Ha
	FILM	CR	0.002	0.0027	−0.002	0.007	−35.76	<0.001	Ha

Sensitivity	LUMIX	ICR	0.035	0.0203	0.001	0.068	−3.21	<0.001	Ha
	LUMIX	CR	−0.017	0.0219	−0.053	0.019	−3.78	<0.001	Ha
	LUMIX	FILM	−0.065	0.0246	−0.105	−0.025	−1.43	0.08	Ho
	ICR	CR	−0.052	0.0210	−0.087	−0.017	−2.28	0.01	Ha
	ICR	FILM	−0.100	0.0256	−0.142	−0.057	−0.02	0.49	Ho
	FILM	CR	0.048	0.0316	−0.004	0.100	−1.66	0.05	Ha

Specificity	LUMIX	ICR	−0.012	0.0192	−0.043	0.020	−4.60	<0.001	Ha
	LUMIX	CR	−0.012	0.0158	−0.038	0.014	−5.59	<0.001	Ha
	LUMIX	FILM	−0.031	0.0182	−0.061	−0.001	−3.79	<0.001	Ha
	ICR	CR	0.000	0.0173	−0.028	0.028	−5.79	<0.001	Ha
	ICR	FILM	−0.019	0.0182	−0.049	0.011	−4.42	<0.001	Ha
	FILM	CR	0.019	0.0124	−0.001	0.040	−6.50	<0.001	Ha

AUC	LUMIX	ICR	0.009	0.0646	−0.098	0.115	−1.412	0.08	Ho
	LUMIX	CR	−0.016	0.0350	−0.073	0.042	−2.408	0.008	Ha
	LUMIX	FILM	−0.026	0.0412	−0.093	0.042	−1.807	0.04	Ha
	ICR	CR	−0.024	0.0660	−0.133	0.084	−1.145	0.13	Ho
	ICR	FILM	−0.034	0.0508	−0.118	0.049	−1.292	0.10	Ho
	FILM	CR	0.010	0.0463	−0.066	0.086	−1.945	0.03	Ha

^a^Each comparison was calculated from 980 observations (70 cases, seven radiologists, and two devices). PPV: positive predictive value, ROC: receiver operating characteristic, AUC: area under ROC curve, SE: standard error of the mean, Ho: null hypothesis, Ha: alternative hypothesis for testing equivalence, *α*: significance of the test (0.05), *δ*: difference of the means allowed to achieve equivalence, *z*: test for difference of compared devices, that is, *z* = (|Difference (*I* − *J*)| − *δ*)/SE, *H*: retained hypothesis equivalence at *δ* level (“Ha” indicates equivalence achieved and “Ho” indicates failing to reject the null hypothes).

Ho: |difference (*I* − *J*)| − *δ* = 0.

Ha: |difference (*I* − *J*)| − *δ* < 0.

**Table 7:** Equivalence tests for accuracy, PPV, sensitivity, specificity, and AUC by paired devices (*δ* = 0.05).

Variable^a^	Compared devices		(1-2*α*)% confidence interval for equivalence testing		*z*	*P*	*H*	*δ*_*e*_
	(*I*) device	(*J*) device	Lower	Upper				
Accuracy	LUMIX	ICR	−0.013	0.034	−2.80	0.003	Ha	0.034
	LUMIX	CR	−0.036	0.008	−2.69	0.004	Ha	0.036
	LUMIX	FILM	−0.072	−0.022	−0.20	0.42	Ho	0.072
	ICR	CR	−0.047	−0.002	−1.84	0.03	Ha	0.047
	ICR	FILM	−0.084	−0.031	0.44	0.67	Ho	0.084
	FILM	CR	0.006	0.060	−1.06	0.14	Ho	0.060

PPV	LUMIX	ICR	−0.011	0.006	−9.10	<0.001	Ha	0.011
	LUMIX	CR	−0.015	0.001	−8.68	<0.001	Ha	0.015
	LUMIX	FILM	−0.017	−0.001	−8.20	<0.001	Ha	0.017
	ICR	CR	−0.012	0.003	−9.68	<0.001	Ha	0.012
	ICR	FILM	−0.013	0.000	−1.71	<0.001	Ha	0.013
	FILM	CR	−0.002	0.007	−17.47	<0.001	Ha	0.007

Sensitivity	LUMIX	ICR	0.001	0.068	−0.76	0.22	Ho	0.068
	LUMIX	CR	−0.053	0.019	−1.49	0.07	Ho	0.053
	LUMIX	FILM	−0.105	−0.025	0.61	0.73	Ho	0.105
	ICR	CR	−0.087	−0.017	0.09	0.54	Ho	0.087
	ICR	FILM	−0.142	−0.057	1.94	0.97	Ho	0.142
	FILM	CR	−0.004	0.100	−0.08	0.47	Ho	0.100

Specificity	LUMIX	ICR	−0.043	0.020	−2.00	0.023	Ha	0.043
	LUMIX	CR	−0.038	0.014	−2.43	0.008	Ha	0.038
	LUMIX	FILM	−0.061	−0.001	−1.05	0.15	Ho	0.061
	ICR	CR	−0.028	0.028	−2.90	0.002	Ha	0.028
	ICR	FILM	−0.049	0.011	−1.68	0.05	Ha	0.049
	FILM	CR	−0.001	0.040	−2.47	0.007	Ha	0.040

AUC	LUMIX	ICR	−0.098	0.115	−0.638	0.26	Ho	0.115
	LUMIX	CR	−0.073	0.042	−0.980	0.16	Ho	0.073
	LUMIX	FILM	−0.093	0.042	−0.592	0.28	Ho	0.093
	ICR	CR	−0.133	0.084	−0.387	0.35	Ho	0.133
	ICR	FILM	−0.118	0.049	−0.308	0.38	Ho	0.118
	FILM	CR	−0.066	0.086	−0.865	0.19	Ho	0.086

^a^Each comparison was calculated from 980 observations (70 cases, seven radiologists, and two devices). PPV: positive predictive value, ROC: receiver operating characteristic, AUC: area under ROC curve, SE: standard error of the mean, Ho: null hypothesis, Ha: alternative hypothesis for testing equivalence, *α*: significance of the test (0.05), *δ*: difference of the means allowed to achieve equivalence, *z*: test for difference of compared devices, that is, *z* = (|Difference (*I* − *J*)| − *δ*)/SE, *H*: retained hypothesis equivalence at *δ* level (“Ha” indicates equivalence achieved and “Ho” indicates failing to reject the null hypothes).

Ho: |difference (*I* − *J*)| − *δ* = 0.

Ha: |difference (*I* − *J*)| − *δ* < 0.

**Table 8:** Evaluation of dense breasts. Mean values, pairwise comparisons, and observed delta for equivalence for TP, TN, FP, sensitivity, specificity, accuracy, VPP, VPN, and AUC.^a^

	TP	TN	FP	FN	SEN	SPE	ACC	VPP	VPN	AUC
Device (resolution)										
CR (3,560 × 4,640)	0.46	0.33	0.05	0.16	0.74	0.86	0.78	0.77	0.53	0.86
ICR (2,436 × 3,636)	0.46	0.30	0.08	0.16	0.74	0.79	0.76	0.76	0.53	0.90
LUMIX (2,538 × 3,463)	0.45	0.29	0.10	0.17	0.73	0.75	0.73	0.73	0.53	0.88
FILM	0.51	0.33	0.05	0.11	0.82	0.88	0.84	0.86	0.53	0.90

Tests of model effects										
Chi-square	26.01	7.25	7.25	9.26	12.70	16.52	24.84	10.23	10.02	4.82
Degree of freedom	3	3	3	3	3	3	3	3	3	3
*P* value	0.026	0.064	0.064	0.026	0.005	0.001	0.000	0.017	0.018	0.186

Pairwise comparisons										
CR versus ICR										
Bonferroni's significance	1.000	1.000	1.000	1.000	1.000	0.944	1.000	0.568	1.000	0.304
Delta for equivalence	0.022	0.061	0.061	0.022	0.036	0.0155	0.068	0.018	0.0	0.073
CR versus LUMIX										
Bonferroni's significance	1.000	0.223	0.223	1.000	1.000	0.063	0.518	0.011	0.424	1.000
Delta for equivalence	0.040	0.073	0.073	0.040	0.065	0.0176	0.093	0.075	0.001	0.049
FILM-CR										
Bonferroni's significance	0.094	1.000	1.000	0.094	0.048	1.000	0.078	0.009	0.380	0.532
Delta for equivalence	0.091	0.026	0.026	0.091	0.0144	0.068	0.0102	0.0134	0.0	0.068
FILM-ICR										
Bonferroni's significance	0.045	0.662	0.662	0.045	0.016	0.449	0.005	0.009	1.000	1.000
Delta for equivalence	0.083	0.069	0.069	0.083	0.0138	0.0172	0.0133	0.0139	0.0	0.038
FILM-LUMIX										
Bonferroni's significance	0.042	0.193	0.193	0.042	0.014	0.046	0.000	0.010	0.147	1.000
Delta for equivalence	0.097	0.084	0.084	0.097	0.0154	0.0197	0.0154	0.0199	0.001	0.052

^a^Each mean was calculated from 147 observations (21 cases and seven radiologists). TP: true positive proportion, TN: true negative proportion, FN: false negative proportion, FP: false positive proportion, PPV: positive predictive value, SEN: sensibility; SPE: specificity; ACC: accuracy, ROC: receiver operating characteristic, AUC: area under ROC curve, *δ*: difference of the means allowed to achieve equivalence (i.e., to claim equivalence with a significant value).
